# Reassessment of diagnostic criteria in cutaneous lymphocytic infiltrates

**DOI:** 10.1590/S1516-31802004000400006

**Published:** 2004-07-01

**Authors:** Ana Cristina Cotta, Maria Letícia Cintra, Elemir Macedo de Souza, Luis Alberto Magna, José Vassallo

**Keywords:** Cutaneous T-cell lymphoma, Pseudo-lymphoma, Mycosis fungoides, Immunohisto-chemistry, Differential diagnosis, Linfoma de Células T Cutâneo, Pseudolinfoma, Micose fungóide, Imunohistoquímica, Diagnóstico diferencial

## Abstract

**CONTEXT:**

Non-specific lymphocytic infiltrates of the skin pose difficulties in daily practice in pathology. There is still a lack of pathognomonic signs for the differential diagnosis between benign and malignant lymphocytic infiltrates.

**OBJECTIVE:**

To evaluate the morphological and immunohistochemical profile of lymphocytic infiltrations of the skin according to clinical outcome.

**TYPE OF STUDY:**

Retrospective; histopathological and immunohistochemical analysis.

**SETTING:**

Referral center, university hospital.

SAMPLE: 28 cases of lymphocytic infiltrates of difficult differential diagnosis selected from the records.

**MAIN MEASUREMENTS:**

Eighteen histological variables and the immunophenotypic profile were assessed using the CD4, CD8, CD3, CD20 and CD30 lymphoid markers and compared to subsequent follow-up.

**RESULTS:**

The most common diagnoses were: initial mycosis fungoides (eight cases) and drug reactions (five cases). Single morphological variables did not discriminate between benign and malignant infiltrates except for the presence of Pautrier-Darier's microabscesses, which were found only in mycosis fungoides (p = 0.015). Patterns of superficial and deep infiltration (p = 0.037) and also the presence of eosinophils (p = 0.0207) were more frequently found in benign lymphocytic infiltrates. Immunohistochemical profile of T-cell subsets showed overlap between benign and malignant infiltrates with a predominance of CD4-positive (helper) lymphocytes in the majority of cases.

**CONCLUSIONS:**

A combination of clinical and histological features remains the most reliable approach for establishing a definite diagnosis in cases of lymphoid skin infiltrates.

## RESUMO

**CONTEXTO:**

Infiltrações linfocitárias não-específicas da pele representam dificuldades diagnósticas na prática diária da patologia. Não há sinais patognomônicos para o diagnóstico diferencial entre infiltrações linfocitárias benignas e malignas.

**OBJETIVOS:**

Avaliar o perfil morfológico e imunofenotípico das infiltrações linfocitárias de acordo com a evolução clínica.

**TIPO DE ESTUDO:**

Retrospectivo: análise histopatológica e imunoistoquímica.

**LOCAL:**

Centro de referência, hospital universitário.

**AMOSTRA:**

28 casos de infiltrações linfocitárias de diagnóstico diferencial difícil selecionados dos arquivos.

**PRINCIPAIS MEDIDAS:**

Análise de 18 variáveis histológicas e perfil imunofenotípico utilizando os marcadores linfóides CD4, CD8, CD3, CD20 e CD30, comparados à evolução clínica.

**RESULTADOS:**

Os diagnósticos mais comuns foram: micose fungóide — inicial (oito casos) e farmacodermias (cinco casos). Variáveis morfológicas isoladas não discriminaram infiltrados benignos e malignos, exceto pela presença dos microabscessos de PautrierDarier, que foram encontrados apenas na micose fungóide (p = 0,015). O padrão de infiltração superficial e profunda (p = 0,037) e a presença de eosinófilos (p = 0,0207) foram mais freqüentes nas infiltrações linfocitárias benignas. O perfil imunoistoquímico dos linfócitos T mostrou sobreposição entre infiltrações benignas e malignas, com predomínio de linfócitos T auxiliares CD4 positivos na maioria dos casos.

**CONCLUSÃO:**

A combinação das informações clínicas e histológicas representa a abordagem mais consistente para o diagnóstico diferencial dos infiltrados linfóides cutâneos.

## INTRODUCTION

Classic textbooks have described five “L” categories for the differential diagnosis of lymphocytic infiltrates of the skin: lymphoma, lymphocytoma cutis, lupus erythematosus, polymorphous light eruption, and Jessner's lymphocytic infiltration of the skin.^1^ Leprosy, syphilis, lichen striatus, necrobiosis lipoidica, bite reactions, and the mnemonic “DRUGS” categories of dermatophytes, reticular erythematous mucinosis, urticarial stages of bullous pemphigoid, gyrate erythemas, localized scleroderma and drug reactions, have been added to the list.^2^ Other differential diagnoses include lichenoid purpura, lichen sclerosus et atrophicus,^3^ multiform erythema and psoriasis.^4^ Ackerman's algorithmic method includes mycosis fungoides 23 times in six different reaction patterns.^5^ According to Diaz-Cascajo^6^ there is no single reliable criterion that allows distinction between inflammation and neoplasia in lymphoproliferative skin disorders.^6^ Distinction between benign and neoplastic skin lymphoid infiltrates is of utmost importance for ensuring adequate therapy and prognostic evaluation.

Two recent publications have shown divergent opinions regarding the role of the immunophenotyping of T-cell subsets as a diagnostic tool for cutaneous lymphocytic infiltrates. Hudson and Smoller^7^ stated that a simple CD4:CD8-positive lymphocyte ratio can be obtained from a cutaneous infiltrate that is predominantly comprised of T-cells and that this ratio can be used to make a diagnosis of mycosis fungoides with a high level of sensitivity and specificity, on the basis of studies made by Izban et al.^8^ Concomitantly, an opposing point of view^9^ has been put forward, in which it is stated that predominance of CD4-positive cells is seen in a wide variety of non-neoplastic conditions and therefore cannot be used to discriminate cutaneous T-cell lymphoma from inflammatory dermatoses. The former opinion is substantiated by some works,^8,10^ and the latter by others.^11,12^ Since no consensus has been reached in the literature, the purpose of the present study was to perform a review of the different causes of lymphocytic infiltrates of the skin via a retrospective study, with emphasis on morphological clues and also the role of immunophenotyping in making appropriate diagnoses.

## METHODS

A retrospective study was made on consecutive cases of cutaneous lymphoid infiltrates of unknown etiology at the Department of Pathology, Universidade Estadual de Campinas (Unicamp), Campinas, São Paulo, Brazil. From 73 cases studied between 1989 and 1999, 28 were selected. The inclusion criteria were a diagnosis of lymphocytic infiltrates of the skin presenting some histopatho-logical findings similar to those described for early patch, plaque or late patch mycosis fungoides,^5^ as well as similarity to histopatho-logical findings of mycosis fungoides variants previously described,^13^ and the availability of detailed clinical files and paraffin blocks for additional immunohistochemical studies. In the cases selected, the definitive diagnosis of mycosis fungoides had been established clinically and histologically, and was further supported by the course of the disease. For patients with mycosis fungoides submitted to several biopsies, the earliest ones were selected. Only patients posing difficulties in the differential diagnosis of lymphoid infiltrates were included. Obvious malignant high-grade non-Hodgkin lymphomas were excluded. Patients suffering from advanced-stage primary nodal lymphomas with clinically evident involvement of the skin were excluded, as well as those with evidence of extracutaneous disease appearing within six months after the diagnosis, in accordance with the criteria of the European Organization for Research and Treatment of Cancer (EORTC).^14^

For each biopsy specimen, slides stained with hematoxylin and eosin were assessed for eighteen histological criteria, in accordance with reaction patterns described by Ackerman and others.^5,15^ These variables, listed in Table 1, were judged to be present or absent and were recorded by two observers who had no clinical information at the time of evaluating these criteria (Maria Letícia Cintra, Ana Cristina Cotta).

**Table 1:** Morphological findings from cutaneous infiltrates of the skin in 28 cases of cutaneous lymphoid infiltrates

Morphologic criteria	Diagnosis						
	Mycosis fungoides		Benign dermatosis		Parapsoriasis		
	n/total	%	n/total	%	n/total	%	p value
Band-like infiltration	5/8	62.5	4/12	33.3	2/8	25.0	0.2631
Psoriasiform hyperplasia	4/8	50.0	8/12	67.7	4/8	50.0	0.6778
Spongiosis	2/8	25.0	0/12	0	1/8	12.5	0.2046
Abnormal cornified layer	6/8	75.0	10/12	83.3	4/8	50.0	0.2614
Superficial perivascular	7/8	87.5	11/12	91.7	8/8	100	0.6104
Interstitial infiltration	8/8	100	12/12	100	7/8	87.5	0.2735
Superficial and deep infiltration	0/8	0	7/12	63.6	4/8	50.0	0.0377
Folliculitis and perifolliculitis	2/8	28.6	7/12	63.6	3/8	37.5	0.2916
Nodular infiltration	1/8	14.3	2/12	16.7	1/8	12.5	0.9665
Disproportionate exocytosis	5/8	62.5	4/12	33.3	4/8	50.0	0.4276
Prominent eosinophils	2/8	25.0	7/12	58.3	0/8	0	0.0207
Aligned lymphocytes	5/8	62.5	7/12	58.3	5/8	62.5	0.9753
Lymphocyte halos	5/8	62.5	6/12	50.0	4/8	50.0	0.8357
Pautrier-Darier's microabscesses	3/8	37.5	0/12	0	0/8	0	0.0150
Atypical lymphocytes	6/8	62.5	7/12	58.3	2/8	25.0	0.2425
Larger intraepidermal	1/8	12.5	3/12	25.0	0/8	0	0.2895
Fibrosis	1/8	12.5	4/12	33.3	3/8	37.5	0.4823
Edema	1/8	12.5	4/12	33.3	1/8	12.5	0.4131

Paraffin-embedded tissue specimens were selected from files and submitted to immunohistochemical studies. All immunophenotypic studies were performed on histological sections of 5 μm in thickness. The antibodies used were CD4 [clone CD45RO/OPD4 (Dako-Patts, Carpentiria, USA); dilution 1:50], CD8 [clone C8/144B (Dako); dilution 1:50], CD3 [polyclonal (Dako); dilution 1:50], CD20 [clone L26 (Dako); dilution 1:100] and CD30 [clone BerH2 (Dako); dilution 1:20]. A steamer was used as the epitope retrieval method and the Envision polymer (Dako) was used as the reaction amplifier.

Two of us (José Vassallo, Ana Cristina Cotta) evaluated the immunohistochemical sections for the percentage of stained cells, without knowledge of the previous diagnosis.

Data were analyzed via the chi-squared and Kruskal-Wallis tests respectively for morphological and immunohistochemical variables. Statistical significance was set at p < 0.05.

## RESULTS

Patients were grouped according to clinical data and follow-up. The groups consisted of: mycosis fungoides (8 cases), benign lymphoid infiltrates (12 cases) and suspected mycosis fungoides (8 cases). The latter category included patients under prolonged follow-up presenting large-plaque parapsoriasis (4 cases), or incomplete clinical and histological findings for mycosis fungoides and also incomplete response to non-destructive therapies (4 cases).^11^

The inflammatory dermatoses found were skin allergies due to drugs (5 cases, one with prominent photosensitivity), pseudo-lymphoma (2 cases, one associated with anti-hypertensive drugs), Jessner's lymphocytic infiltration, neurodermatitis, prurigo nodularis, lupus erythematosus, and erythema annulare centrifugum (1 case each).

**Figure 1:**
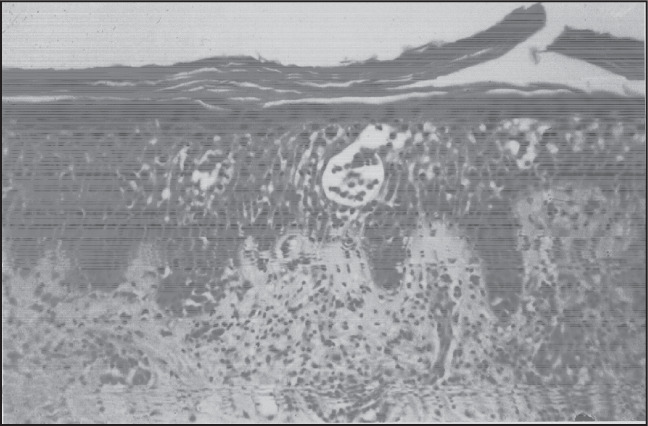
Mycosis fungoides: compact orthokeratosis, Pautrier-Darier's microabscess, moderate spongiosis (hematoxylin-eosin; 200 x).

From the eighteen morphological criteria studied, Pautrier-Darier's microabscesses were present only in some of the mycosis fungoides patients (3 out of 8 cases; 37.5%) (Figure 1). Superficial and deep lymphocytic infiltrates were less common among mycosis fungoides patients and more prominent in benign infiltrates (p = 0.037). The most frequent epidermal reaction pattern was psoriasiform hyperplasia, which was present in about half of cases. There was compact hyperkeratosis, or parakeratosis, in 75% of the mycosis fungoides cases, but this finding was also common in the groups with inflammatory dermatosis and suspected lymphoma. Absence of spongiosis was not a prominent feature in mycosis fungoides, in comparison with the other groups. Superficial perivascular patterns combined with interstitial patterns of infiltration were present in the majority of patients in all groups and band-like subepidermal infiltration was present in more than half of the mycosis fungoides patients. Prominent exocytosis with scant spongiosis was more frequent in the lymphoma and suspected lymphoma (62.5% and 50.0%) cases than in the benign infiltrate cases (30.8%). The presence of eosinophils was more common in the group of benign lymphocytic infiltrates (p = 0.0207) (Table 1).

The immunohistochemical studies demonstrated the presence of a predominant T-cell immunophenotype in all the cases selected, with less than 30% B-cells in all cases, except for one patient with suspected cutaneous lymphoma.^16^ CD30 was negative except for two mycosis fungoides cases.

**Figure 2:**
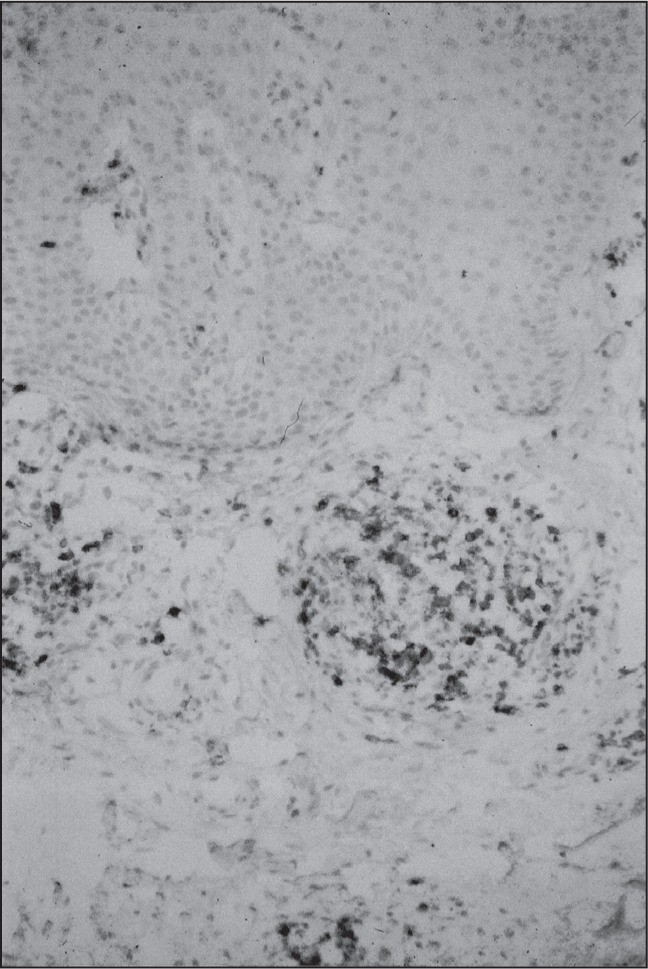
Benign lymphocytic infiltration of the skin: CD4-positive lymphoid cells (immunoperoxidase; 200 x).

**Figure 3:**
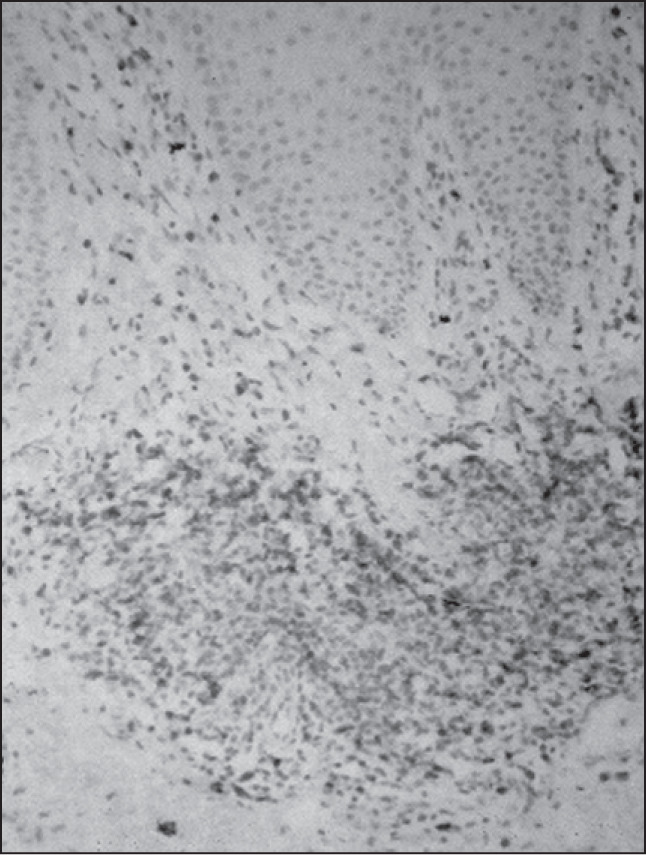
Mycosis fungoides: CD8-positive infiltrating lymphocytes (immunoperoxidase; 200 x).

There was prominent helper/inducer T-cell (CD4-positive) predominance (Figure 2), in comparison with suppressor/cytotoxic (CD8-positive) stained cells, for all groups. The CD4:CD8 ratio was about 4. CD4 lymphocytes constituted about 80% of the infiltrating lymphocytes in all groups. Statistical analysis did not show significant differences between the groups in relation to the CD4/CD8 ratio. There was one mycosis fungoides case with predominance of CD8 lymphocytes (Figure 3 and Table 2).

**Table 2:** Immunohistochemical studies of 28 cases of cutaneous lymphoid infiltrates

Mycosis fungoides	CD4 (%)	CD8 (%)	CD3 (%)	CD30 (%)	CD20 (%)
Median	80	20	85	0	10
Mean	72.2	24.4	82.5	7.5	11.2
SD	23.9	25	12.8	17.5	9.9
Min	30	1	60	0	0
Max	100	80	100	50	30
**Benign dermatosis**					
Median	80	20	90	0	10
Mean	75.4	23.9	73.1	0	10
SD	24.5	25.6	30.9	0	9.1
Min	30	1	10	0	0
Max	100	80	100	0	30
**Parapsoriasis**					
Median	80	30	90	0	5
Mean	76.3	37.5	73.8	0	13.8
SD	18.5	24.9	23.3	0	27.2
Min	40	10	40	0	0
Max	100	80	90	0	80
p value	0.9910	0.2403	0.8563	-	0.6412

*SD = standard deviation; Min = minimum, Max = maximum.*

## DISCUSSION

There is a large group of skin diseases that are characterized by increased numbers of lymphocytic cells. For therapeutic purposes, benign hyperplasia needs to be distinguished from neoplastic conditions. In our material, the most difficult differential diagnosis was between drug reactions and cutaneous T-cell lymphomas. Both are more frequent in older patients undergoing chronic treatments, especially with anti-hypertensive drugs, and they are also described in the etiology of some pseudolymphomas.^11^ Another important aspect that must be considered in relation to drug reactions is their clinical presentation as erythroderma simulating Sézary Syndrome. The differential diagnosis for exfoliative erythroderma is always difficult and may not be established in about 30% to 40% of such patients.^17^ This includes atopic dermatitis, psoriasis, pityriasis rubra pilaris, contact dermatitis, seborrheic dermatitis, pemphigus foliaceous, leukemia and other internal malignancies.^17^ In fact, it may be necessary to take several biopsies in order to detect definite signs of cutaneous lymphoma. Lupus erythematosus and leprosy cases are frequent in our daily routine, but only one case presented histological findings allowing differential diagnosis with mycosis fungoides.

Among the morphological variables that were evaluated, Pautrier-Darier's microabscesses were present only in mycosis fungoides cases and were absent in the other groups. Although 100% specific for this group of patients, Pautrier-Darier's micro-abscesses were present only in 37.5% of our mycosis fungoides cases. The high specificity and low sensitivity of Pautrier-Darier's microabscesses for mycosis fungoides that we found is in agreement with previous studies reporting frequencies from 4.2% to 37.5%.^4,15,18,19^ Prominent eosinophils were more frequent within benign lymphoid infiltrates. This may reflect the predominance of drug reaction cases in this group. The pattern of superficial and deep perivascular infiltration was more commonly seen in benign lymphocytic infiltrations and suspected lymphoma cases.

The mycosis fungoides patients had been submitted to several biopsies. Disease progression is related to deeper infiltrates but we studied just the initial biopsy specimens. Prominent atypical lymphocytes did not discriminate for mycosis fungoides. Cerebriform lymphocytes are usually considered to be discriminating variables^4^ for the diagnosis of mycosis fungoides, but they were not prominent in the sections we studied. This was perhaps because we used the early mycosis fungoides biopsies in the cases when multiple biopsies had been performed. The low percentage of spongiosis within the benign infiltrate group may be explained by the chronic nature of the selected cases in this sample. Superficial, deep and nodular infiltrate and folliculitis could not be evaluated in two cases of mycosis fungoides and one of benign lymphoid infiltration due to scant reticular dermis, and the absence of adnexa in one case.

One mycosis fungoides case had lymphocytes that were 50% CD30-positive, but this did not change the diagnosis to CD30-positive large T-cell lymphoma. CD30 should be expressed by the majority (> 75%) of neoplastic cells, in a consistent morphological context, to be included in this group.^14,20^ The absence of CD30 positivity in the other two groups does not characterize CD30 as a marker for malignancy, as it may be encountered in non-malignant lymphoproliferative disorders^6,20,21^ and even in association with non-lymphoproliferative entities.^22^

Two studies published in 1999 took opposing points of view concerning the role of the immunophenotyping of cutaneous infiltrates.^7,9^ A high CD4:CD8 ratio was regarded as both sensitive and specific for the diagnosis of mycosis fungoides by Hudson and Smoller,^7^ on basis of an earlier study by Izban et al.^8^ In that study,^8^ 35 biopsies from 29 mycosis fungoides patients were evaluated. They found a CD4:CD8 ratio of more than 2:1 in 31 of the 35 sections, but no control group results were reported in that study. Bakels et al.^23^ performed a semiquantitative estimation of CD8-positive cells in frozen sections. They found a greater admixture of CD8-positive small lymphocytes in 11 pseudolymphoma cases than in 9 mycosis fungoides cases. No statistically significant difference between the groups was described.^23^

Nuckols et al.^10^ also reported that a high CD4:CD8 ratio was a helpful tool in the differential diagnosis between mycosis fungoides and inflammatory conditions. They presented a control group composed of spongiosis and lichenoid dermatitis patients. Their results indicated statistically significant differences between the groups but only for intraepidermal lymphocytes. The CD4:CD8 ratio in the dermis did not show any difference (p = 0.18), and about 95% of the counted cells were in the dermis. This included about 9.6 CD4 cells and 5.3 CD8 cells in the epidermis versus 250.2 CD4 cells and 124.8 CD8 cells in the dermis. Therefore, we should consider that this finding is much more related to the subset of lymphocytes that contribute to the phenomenon of epidermotropism than to the CD4:CD8 ratio itself. The reason why the mean observed was so high was mostly because of two mycosis fungoides cases with prominent epidermotropism. An estimate of the proportion of intraepidermal lymphocytes was made for the cases in the present study, but the number of cells detected was too small to allow adequate statistical analysis and did not differ from the CD4-CD8 subtype of T-cells identified in the corresponding dermis (data not published).

On the other hand, Glusac et al.^9^ believed that predominance of CD4 positive cells is seen in a wide variety of non-neoplastic conditions and cannot be used as a discriminatory parameter for the differential diagnosis between cutaneous T-cell lymphomas and inflammatory dermatoses. Some authors also regard the CD4:CD8 ratio as a nonspecific finding, given the existence of cases of mycosis fungoides with predominant CD8.

## CONCLUSIONS

Our results corroborate previous data in which it was concluded that the immunophenotypic profile must be considered with caution because benign lymphocytic infiltrates^9,11^ as well as small plaque parapsoriasis biopsy specimens^12^ may display predominant CD4 expression. Definite diagnosis still needs clinicopathological correlation and careful follow-up.
